# The Lysine Demethylase KDM7A Regulates Immediate Early Genes in Neurons

**DOI:** 10.1002/advs.202301367

**Published:** 2023-08-10

**Authors:** Yifan Wang, Qin Hong, Yueyue Xia, Zhao Zhang, Bo Wen

**Affiliations:** ^1^ Key Laboratory of Metabolism and Molecular Medicine of Ministry of Education, Department of Biochemistry and Molecular Biology, School of Basic Medical Sciences Fudan University 200032 130 Dong An Road Shanghai China; ^2^ Shengli Clinical Medical College of Fujian Medical University, Center for Experimental Research in Clinical Medicine Fujian Provincial Hospital 134 East Street Fuzhou 350001 China

**Keywords:** histone demethylases, histone modification, immediate early gene, KDM7A, N2a differentiation

## Abstract

Lysine demethylase KDM7A removes histone modifications H3K9me1/2 and H3K27me1/2. KDM7A plays critical roles in gene expression and contribute to biological processes including tumorigenesis, metabolism, and embryonic development. However, the functions of KDM7A in mammalian nervous system are still poorly explored. In this study, functional roles of KDM7A are comprehensively investigated in neuronal cells by applying CUT&Tag‐seq, RNA‐seq and mice models. Knockdown of *Kdm7a* in N2A cells result in the alteration of histone modifications near transcription start sites (TSSs) and the expression changes of a large number of genes. In particular, the expression of immediate early genes (IEGs), a series of genes maintaining the function of the nervous system and associating with neurological disorders, are significantly decreased upon *Kdm7a* knockdown. Furthermore, in vivo knockdown of *Kdm7a* in dentate gyrus (DG) neuron of mice hippocampus, via Adeno‐associated virus (AAV)‐based stereotaxic microinjection, led to a significant decrease of the expression of c‐Fos, a marker of neuron activity. Behavior assays in mice further revealed that *Kdm7a* knockdown in hippocampus repress neuron activity, which leading to impairment of emotion and memory. Collectively, the study reveals that KDM7A affects neuron functions by regulating IEGs, which may provide new clues for understanding epigenetic mechanisms in neurological disorders.

## Graphical abstract

This study reveals that KDM7A targets thousands of genes, affects a variety of histone modifications, and regulates gene expression probably by collaborating with TFs. Particularly, KDM7A regulates the expression of IEGs, which play important roles in neuronal differentiation and activity.

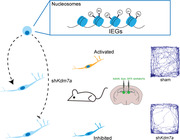

## 1 Introduction

Neuronal loss in the central nervous system leads to cognitive impairment, which is the most prominent feature ofneurodegenerative diseases.^[^ ^1^ ^]^ The prevalence and incidence of neurodegenerative diseases increase dramatically with age.^[^ ^2^ ^]^ As the world's population ages, the number of people suffering from these diseases will increase, which will result in substantial pressure on society and families.^[^ ^3^ ^]^ Epigenetic processes are involved in many neurodegenerative diseases, such as Alzheimer's disease,^[^ ^4^ ^]^ Huntington's disease,^[^ ^5^ ^]^ and Parkinson's disease.^[^ ^6^ ^]^ These studies provide opportunities for the availability of drugs and other genetic tools to modify epigenetic changes in the brain. To date, histone deacetylases (HDAC), have long been studied as potential targets for the treatment of neurodegenerative diseases.^[^ ^7^ ^]^ Since the majority of therapies have focused on targeting histone acetylation proteins, it is urgent to find new epigenetic targets.

The KDM7 family consists of three members: KIAA1718 (KDM7A), PHF8 (KDM7B) and PHF2 (KDM7C). These proteins contain ≈1000 amino acids, including a plant homeodomain (PHD) and a Jumonji‐C (JmjC) domain at their N‐terminus.^[^ ^8^ ^]^ As a demethylase, KDM7A is mainly responsible for removing H3K9me1/2 and H3K27me1/2 on histones.^[^ ^9^ ^]^ Some studies demonstrated that KDM7 family proteins have rigid linkers and extended N‐terminals, and once their PHD domain binds with H3K4me3, it cannot remove H3K9me2.^[^ ^8^ ^]^ However, H3K4me3 may stimulate the demethylation of H3K9me2/1, H3K27me2/1, and H4K20me1 on the tail of adjacent histones because it anchors the KDM7 enzyme nearby.^[^ ^8^ ^]^ These KDM7 proteins create a more open chromatin environment at the promoter as a result of their binding with H3K4me3 and the removal of H3K9me2/1, H3K27me2/1 or H4K20me.^[^ ^8^ ^]^ A series of studies reported that KDM7A plays a crucial role in brain development, promotes ES differentiation, tectum development, and neural induction in early embryos.^[^ ^9^, ^10^ ^]^ However, the regulatory mechanism of KDM7A in mammalian nervous system is still unclear.

Immediate early genes (IEGs) are rapidly and transiently expressed in response to external stimuli, therefore, they are widely used as markers of neuron activity.^[^ ^11^ ^]^ The prefrontal cortex and hippocampus play critical roles in cognition, memory and motivation.^[^ ^12^ ^]^ The expression of IEGs has been reported increased in response to social interaction and social recognition in these brain regions.^[^ ^11^, ^13^ ^]^ IEGs are involved in a variety of biological functions, including cell proliferation, differentiation, and synapse formation. IEGs are first upregulated at the transcriptional level in the first round of response to a stimulus before any protein is synthesized, which is essential for the formation of neural circuits, connections between synapses, and consolidation of memories. It's reported that increased DNA methylation and decreased histone acetylation at the promoter of neuronal IEGs are associated with decline in their expression and memory consolidation.^[^ ^14^ ^]^ The H3K9me3 level was found increased at the promoter of neuronal IEGs during aging.^[^ ^15^ ^]^

Our previous study found that KDM7A was expressed in different mouse tissues, including the brain.^[^ ^16^ ^]^ KDM7A is abundantly expressed in brain slices, indicating that it could be relevant to mammalian brain function. In this study, we showed that KDM7A modified H3K9me2 and H3K27me2 as a demethylase at promoters of thousand genes and might collaborate with transcription factors (TFs) to regulate expression of critical IEGs (e.g. *Egr1* and *Atf3*). Dysregulation of IEGs affected the differentiation of neuronal cells and mouse neuronal activity, which led to emotion and memory impairment. We explored the role of KDM7A in the mammalian nervous system, partially elucidated the relevant regulatory mechanisms, and provided insights for research community of neurological diseases.

## 2 Results

### 2.1 Expression of KDM7A is Increased During Neuronal Cells Differentiation

**Figure 1:**
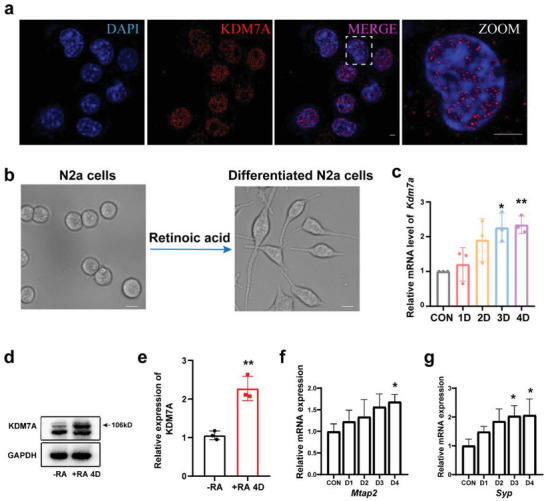
KDM7A increased during differentiation in neuronal cells. a) Representative IF staining in N2a cells. Scale bar, 5 µm. b) Bright field images of N2a cells without or with RA induction for 4 days. Scale bar, 5 µm. c) qRT‒PCR and d, e) WB analysis of KDM7A expression after RA induction (n = 3). The data were presented as the mean ± Standard Error of Measurement (SEM). One‐way ANOVA with Dunnett's multiple comparisons test, *p < 0.05; **p < 0.01; T test: **p < 0.005. f, g) qRT‒PCR was used to detect *Mtap2* and *Syp* expression levels during N2a differentiation (n = 3). The data were presented as the mean ± SEM. One‐way ANOVA with Dunnett's multiple comparisons test, *p < 0.05.

Neuro‐2a (N2a) is a mouse neural crest‐derived cell line that has been widely used to study neuritegrowth and neuronal differentiation.^[^ ^17^ ^]^ KDM7A was highly expressed in the nucleus of N2a cells, as shown by Immunofluorescence (IF) (**Figure** **1**a). To assess the potential function of KDM7A in mammalian nervous system, we selected N2a cells as in vitro model.

First, considering that N2a cells can be induced to differentiate, DMEM containing 2% FBS, and 10 µM Retinoic acid (RA) was utilized to induce N2a cell differentiation. Neuron‐like morphological changes were observed after retinoic acid treatment. We defined cells with neurites at least 1.5 times the length of the cell body diameter as differentiated cells (Figure 1b). After 4 days of induction, KDM7A expression increased in a time‐dependent manner during N2a differentiation (Figure 1c). The expression of KDM7A significantly increased at both mRNA and protein levels (Figure 1c–e) after differentiation. MAP2 is a protein involved in stabilizing microtubules,^[^ ^18^ ^]^ while SYNAPTOPHYSIN is a protein involved in the regulation of short‐term and long‐term synaptic plasticity.^[^ ^19^ ^]^ These proteins are widely used as markers for mature neurons. During N2a differentiation, *Mtap2* and *Syp)* were found to increase in a time‐dependent fashion (Figure 1f,g), indicating that the induction system was successful. Thus, *Mtap2* and *Syp* were used to evaluate the differentiation progress of N2a cells in later analyses.

### 2.2 KDM7A Acts as Key Factor in Differentiation of Neuronal Cells

**Figure 2:**
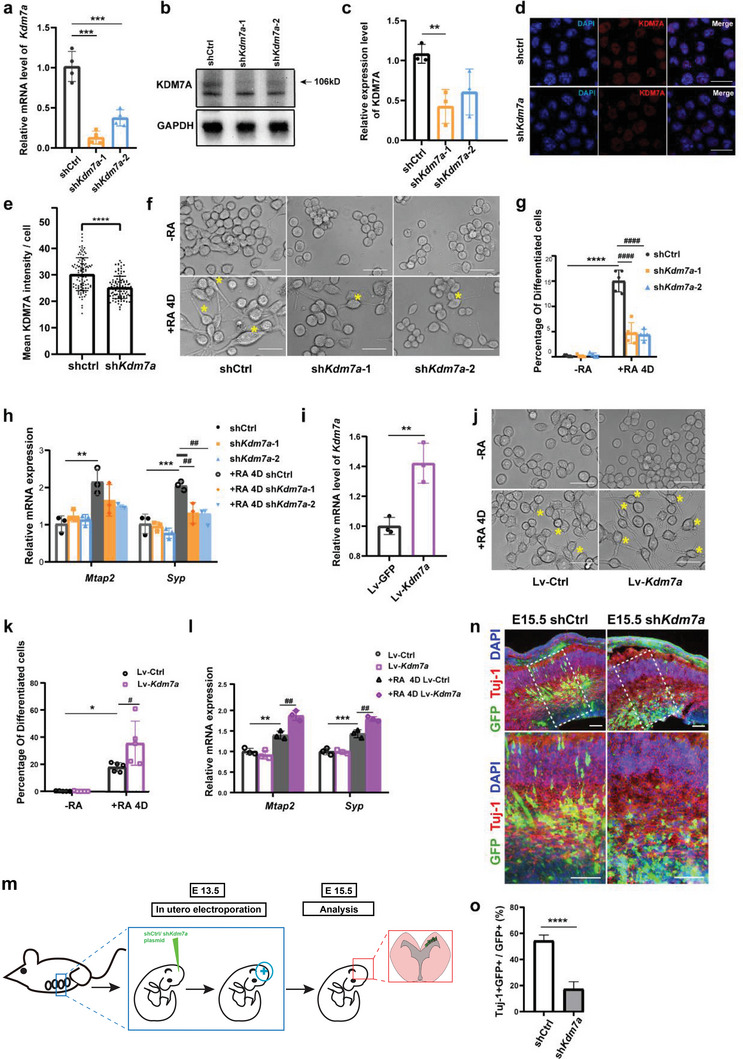
KDM7A promoted neuronal cells differentiation. a) qRT‒PCR and b, c) WB were performed to detect the efficiency of sh*Kdm7a* (n = 3). The data were presented as the mean ± SEM. One‐way ANOVA with Dunnett's multiple comparisons test, **p < 0.01; ***p < 0.00. d, e) IF and quantification of KDM7A in shCtrl and sh*Kdm7a* N2a cells. Florescence intensity was quantified by LAS AF Lite Software (Leica, UK) in 100 cells each group. The data were presented as the mean ± SEM. T test: ****p < 0.0001. Scale bar, 10 µm. f) Representative bright field images of the shCtrl and sh*Kdm7a* cells with or without RA induction. Arrows indicate differentiated cells. Scale bar, 50 µm. g) Quantification of the ratio of differentiated cells. At least 5 fields were quantified for each group. The data were presented as the mean ± SEM. One‐way ANOVA with Sidak's multiple comparisons test, ****p < 0.0001, ####*p* < 0.0001. h) qRT‒PCR detected *Mtap2* and *Syp* expression levels after RA induction in shCtrl and sh*Kdm7a* N2a cells (n = 3). The data were presented as the mean ± SEM. One‐way ANOVA with Dunnett's multiple comparisons test for each gene, **p < 0.01, ***p < 0.001, ##*p* < 0.01. i) qRT‒PCR detected the efficiency of *Kdm7a* overexpression (n = 3). The data were presented as the mean ± SEM. T test: **p < 0.005. j) Representative bright field images of the lv‐Ctrl and Lv‐*Kdm7a* cells with or without RA induction. Arrows indicate differentiated cells. Scale bar, 50 µm. k) Quantification of the ratio of differentiated cells. At least 5 fields were quantified for each group. The data are presented as the mean ± SEM. One‐way ANOVA with Turkey's multiple comparisons test, *p < 0.05, #*p* < 0.05. l) qRT‒PCR detected *Mtap2* and *Syp* expression levels after RA induction in lv‐Ctrl and Lv‐*Kdm7a* cells (n = 3). The data were presented as the mean ± SEM. One‐way ANOVA with Dunnett's multiple comparisons test for each gene, **p < 0.01, ***p < 0.001, ##*p* < 0.01. m) Graphic overview of intrauterine electroporation. n) Representative IF staining of embryonic mouse brain slices. Three brains were analyzed for each group. Scale bar, 200 µm. o) Quantification of Tuj‐1+ GFP+ cells versus GFP+ cells. The data were presented as the mean ± SEM. T test: ****p < 0.0001.

To assess the role of KDM7A in N2a differentiation, we knocked down *Kdm7a* by two shRNA lentiviruses. The knockdown efficiency was detected at both the mRNA and protein levels (**Figure** **2**a–c). IF experiments and the quantification with >100 cells further confirmed the decrease of KDM7A after knockdown (Figure 2d,e). Cells were then induced to differentiation by RA. We counted differentiated cells in the shCtrl and shK*dm7a* N2a cells after treatment with RA for 4 days. Bright fields were randomly chosen and at least 500 cells were counted and quantified (Figure 2f). The percentage of differentiated cells was significantly decreased in the sh*Kdm7a* N2a cells (Figure 2g). The mRNA levels of *Mtap2* and *Syp* were also decreased in the sh*Kdm7a* N2a cells (Figure 2h), suggesting sh*Kdm7a* suppressed the differentiation of N2A cells. To further confirm the effect of KDM7A on N2a differentiation, we overexpressed *Kdm7a* (Figure 2i) and found the differentiation of N2a cells was markedly promoted (Figure 2j–l). These results suggested important functions of KDM7A in neuronal cell differentiation.

Furthermore, we explored the effect of KDM7A on neuron differentiation in vivo. Neuronal development in the mouse cortex begins at E12, and radial glial cells (RG) proliferate in large numbers and begin to migrate to the cortex and differentiate into neurons. Intrauterine electroporation (IUE) is an important technique to study the molecular mechanisms of cell proliferation, differentiation, migration, and maturation in neurodevelopment.^[^ ^20^ ^]^ At 13.5 days of the mouse embryonic stage, the sh*Kdm7a* or shCtrl plasmid was injected into the lateral ventricle of the fetal mouse by IUE. The plasmid was transferred into the cells near the ventricle, and then the embryo was put back, developed for two days, and removed for analysis at the embryonic stage of 15.5, giving rise to the expression of GFP in RG, with GFP remaining after RG differentiation into intermediate progenitors and neurons (Figure 2m).^[^ ^21^ ^]^ Immunofluorescence staining with the neuronal marker Tuj‐1 showed that the labeled cells in the control group began to migrate and differentiate toward the cortex, while the cells of the knockdown group accumulated at the wall of the ventricles (Figure 2n,o). That highlighted the crucial role of KDM7A in neuronal cell differentiation in vivo.

### 2.3 The Potential Mechanism of KDM7A in Mediating Gene Expression

**Figure 3:**
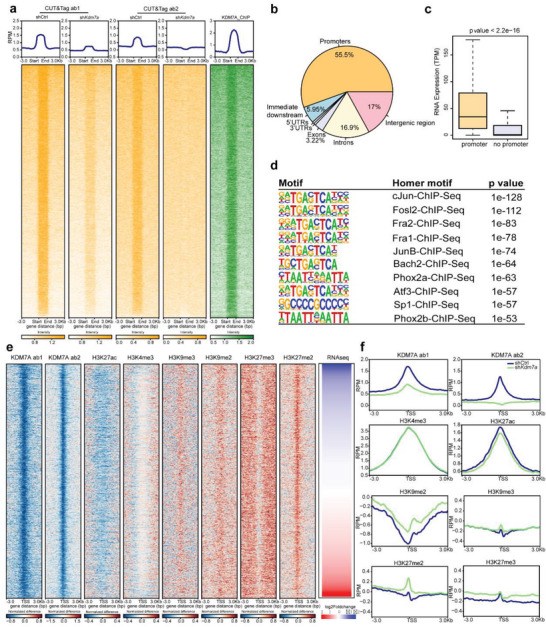
Genome‐wide KDM7A binding and its effect on histone modifications. a) Heatmap of KDM7A by CUT&Tag‐seq (yellow) using two antibodies and ChIP‐seq (green). The rank of chromatin regions (y axis) for CUT&TAGseq and ChIPseq were consistent in heatmap. b) Pie chart represented the characteristics of KDM7A binding on genome. c) Boxplot showed the expression level of genes with KDM7A binding on the promoter or without KDM7A binding. d) The top ten binding motifs of KDM7A throughout genome with CUT&Tag‐seq data. e) Alteration of KDM7A binding density, and histone modifications including H3K9me2, H3K9me3, H3K27me2, H3K27me3, and H3K27ac between shCtrl and sh*Kdm7a* groups. H3K9me2 was detected by ChIP‐seq, and the other five histone modifications were detected by CUT&Tag‐seq. f) Profile of alteration of KDM7A, H3K9me2, H3K9me3, H3K27me2, H3K27me3, and H3K27ac near TSSs.

To investigate the potential regulatory mechanism, we mapped genome‐wide KDM7A binding with ChIP‐seq and CUT&Tag‐seq, which detected similar regions that enriched for KDM7A binding (**Figure** **3**a). Notably, we conducted CUT&Tag‐seq with two KDM7A antibodies and obtained consistent results (Figure 3a). By considering consistent peaks detected by both antibodies, we identified 10,886 KDM7A binding regions, and 55.5% of them located on the promoters (Figure 3b), indicating KDM7A may affect expression of these genes. Indeed, genes with KDM7A binding to the promoter showed significantly higher expression levels than other genes (Figure 3c). We further investigated the binding motifs of KDM7A throughout genome (Figure 3d) and identified 81 motifs. Five of the top ten motifs (sorted by p value) were with similar sequences and could be bound by c‐Jun, Fra2, Fra1, JunB and Atf3, which all belong to the activating protein‐1 (AP‐1) family. One of the major functions of AP‐1 is to activate transcription of a variety of genes involved in cell proliferation, differentiation and neuronal transduction.^[^ ^22^ ^]^ This result may partially explain the higher expression level of genes with KDM7A binding.

Since KDM7A is known as a histone demethylase that specifically abolishes the di‐methylation of H3K9 and H3K27,^[^ ^9^ ^]^ ChIP‐seq and CUT&Tag‐seq were performed to detect the changes of six histone modifications on TSS after *Kdm7a* knockdown. Our results showed that H3K9me2 and H3K27me2 increased at the TSS after *Kdm7a* knockdown, and H3K9me3 showed a slight increase at the TSS (Figure 3e,f). Interestingly, H3K27ac slightly decreased at the TSS after *Kdm7a* knockdown, which may provide a less open chromatin structure at the promoter. These results indicated that some repressive histone modifications increased and active histone modifications decreased at TSS when *Kdm7a* knockdown, which could contribute to the regulation of target genes.

**Figure 4:**
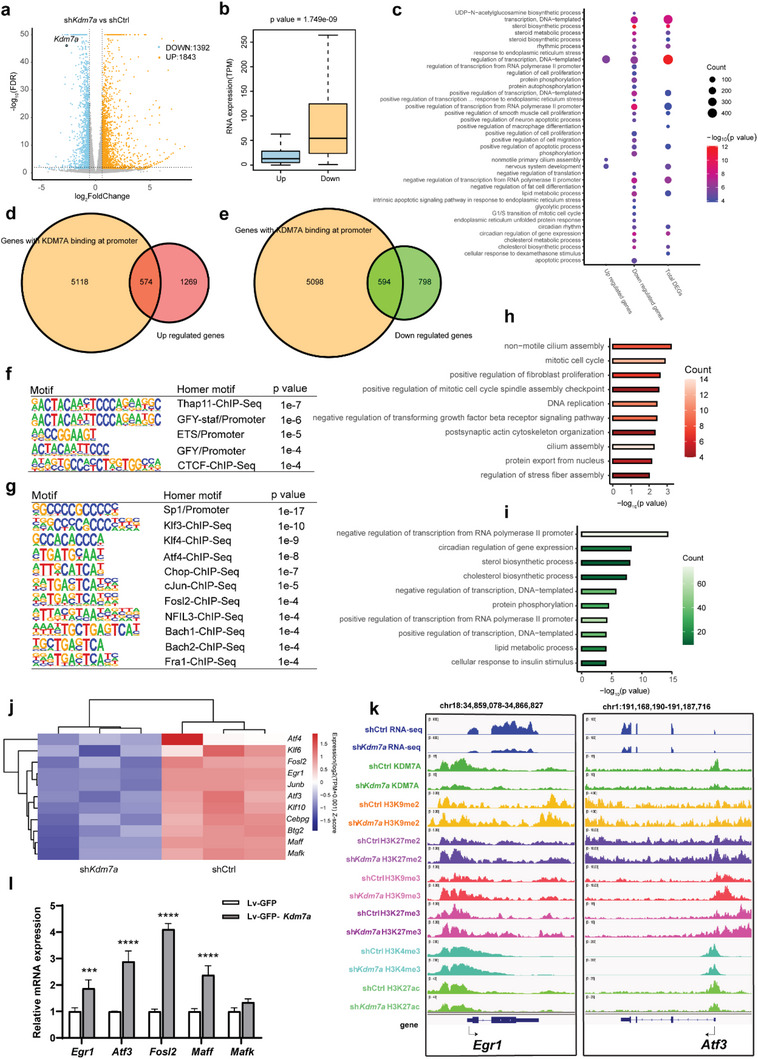
KDM7A regulates the expression of targeted genes. a) Volcano plot illustrated up‐ and down‐regulated genes in the sh*Kdm7a* N2a cells compared with the shCtrl N2a cells. b) Boxplot showed the baseline expression levels of genes up‐ and down‐regulated in shCtrl group. c) GO enrichment analysis of upregulated genes, downregulated genes and total DEGs. The dot size shows the gene count for each hallmark. d) Venn diagram of genes with KDM7A binding to the promoter overlapped with genes up‐regulated after *Kdm7a* knockdown. e) Venn diagram of genes with KDM7A binding to the promoter overlapping with genes down‐regulated after *Kdm7a* knockdown. f) The binding motifs of 574 up‐regulated genes found in (d). g) The binding motifs of 594 down‐regulated genes found in (e). h) GO enrichment analysis of 574 up‐regulated genes found in (d). i) GO enrichment analysis of 594 down‐regulated genes found in (e). j) Heatmap of down‐regulated IEGs with KDM7A binding to promoter. k) Integrative genome visualization (IGV) tracks illustrated RNA‐seq, KDM7A binding site and histone modifications sites at selected gene loci. l) qRT‒PCR detected IEG expression levels when *Kdm7a* was overexpressed in N2a cells (n = 3). The data were presented as the mean ± SEM. Two‐way ANOVA with Sidak's multiple comparisons tests, ***p < 0.001, ****p < 0.0001.

Next, RNA‐seq analysis was performed in shCtrl and sh*Kdm7a* N2a cells. There were 3,235 genes with significant expression changes after *Kdm7a* knockdown, including 1,843 up‐regulated genes (57%) and 1,392 down‐regulated genes (43%) (**Figure** **4**a). Interestingly, up‐regulated genes had a lower expression level compared with down‐regulated genes in shCtrl cells (Figure 4b). Gene Ontology (GO) analysis showed that both upregulated and downregulated genes were mainly enriched in expression regulation (Figure 4c). We further integrated differentially expressed gene (DEG) with genes whose promoters bound by KDM7A. The results showed that 574 genes with KDM7A binding to the promoter were upregulated (Figure 4d), while 594 genes with KDM7A binding to the promoter were downregulated (Figure 4e). The Nearly same amount of up‐ and down‐regulated genes associated with KDM7A binding suggested a complex mechanism of KDM7A on gene regulation.

Furthermore, the binding motifs of these up‐ or down‐regulated genes were identified (Figure 4f,g). The top motif of up‐regulated genes was Thap11, a repressive transcription factor,^[^ ^23^ ^]^ while the top motif of down‐regulated genes was Sp1, an active transcription factor.^[^ ^24^ ^]^ In addition, GO analyses were performed with these up/down‐regulated genes, respectively (Figure 4h,i). Interestingly, many known IEGs were contained in terms negative/positive regulation of transcription from the RNA polymerase II promoter and negative/positive regulation of transcription, DNA‐templated. These IEGs significantly decreased when *Kdm7a* knockdown (Figure 4j). Here, we showed the tracks of two IEGs, *Egr1*, and *Atf3* (Figure 4k). RNA‐seq indicated that the expression of these genes decreased after *Kdm7a* knockdown. The KDM7A peak was located at the promoter region of *Egr1* and *Atf3. Kdm7a* knockdown increased H3K9me2, H3K9me3, H3K27me2 and H3K27me3 levels but decreased H3K27ac levels at the TSS of *Egr1* and *Atf3*. Other IEGs showed similar patterns as shown by the tracks (FigureS1a, Supporting Information). Next, we overexpressed *Kdm7a* in N2a cells and found that IEGs were activated to varying degrees (Figure 4l), which further indicated that *Kdm7a* regulates the expression of these IEGs. All these results suggested that KDM7A may mediate IEG expression by modulating repressive or activating histone modifications at their TSS. Furthermore, our data implied that KDM7A may collaborate with different TFs to activate or repress its targeted genes.

### 2.4 Kdm7a Knockdown in Hippocampal Neurons Affects Mouse Cognition and Emotional Behavior

**Figure 5:**
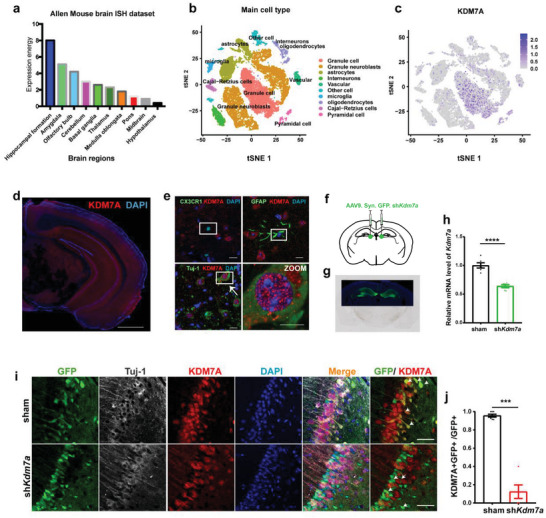
Knockdown of *Kdm7a* in mice brain. a) Expression of *Kdm7a* across brain regions. b, c) Expression of *Kdm7a* across cell types in hippocampus based on scRNA‐seq dataset. d) Representative IF staining in mouse brains. Scale bar, 2 mm. e) Representative IF stainings of KDM7A with different cell types. Scale bar, 10 µm. f) Schematic diagram of stereotaxic microinjection of AAV. g) Representative brain slices show GFP expression 21 days after AAV injection. h) qRT‒PCR analysis of the efficiency of *Kdm7a* knockdown in the mouse hippocampus. (n = 6). The data were presented as the mean ± SEM. T test: ****p < 0.0001. i) Representative IF stainings in the brains of the sham and sh*Kdm7a* mice. Arrows indicate GFP+ KDM7A+ cells in sham or GFP+ KDM7A‐ cells in the sh*Kdm7a* group. Scale bar, 50 µm. j) Quantification of the ratio of KDM7A+GFP+/GFP+ cells (sham n = 7, sh*Kdm7a* n = 5). The data were presented as the mean ± SEM. T test: ***p < 0.001.

We further asked whether altered expression of KDM7A in mice brain would affect mouse cognition and emotional behavior. We first characterized the expression of KDM7A across brain regions ^[^ ^25^ ^]^ and found that expression of *Kdm7a* is the highest in hippocampus compared with others (**Figure** **5**a). We also investigated the expression of *Kdm7a* across cell types in hippocampus based on the scRNA‐seq dataset,^[^ ^26^ ^]^ and observed that KDM7A has the highest expression level in granule cells (one type of neurons) than any other cell type (Figure 5b,c).

Furthermore, IF of KDM7A was achieved in mouse brain slices and showed that KDM7A was expressed in multiple regions (Figure 5d). We assessed KDM7A expression in neuron, microglial and astrocyte cells by co‐immunofluorescence of KDM7A with Tuj‐1 (marker for neurons), CX3 (marker for microglial cells) or GFAP (marker for astrocyte cells). The results showed that KDM7A mainly expressed in neurons marked by Tuj‐1 (Figure 5e), which was consistent with the scRNA‐seq data.

In the hippocampus, a center of declarative memory formation, rapid transcription of IEGs occurs during hippocampal‐dependent learning paradigms, including the Morris water maze, novel environment exposure, and contextual fear conditioning.^[^ ^27^ ^]^ Granule cells are intrinsic excitatory neurons that are abundant in hippocampus and play a crucial role in memory function.^[^ ^28^ ^]^ Thus, we wondered how KDM7A affects the function of neurons in hippocampus. We knocked down neuronal *Kdm7a* in the mouse hippocampus by neuron‐specific Adeno‐associated virus (AAV) stereotaxic injection (Figure 5f,g), which successfully knocked down the expression of *Kdm7a* in hippocampal neurons (Figure 5h–j).

**Figure 6:**
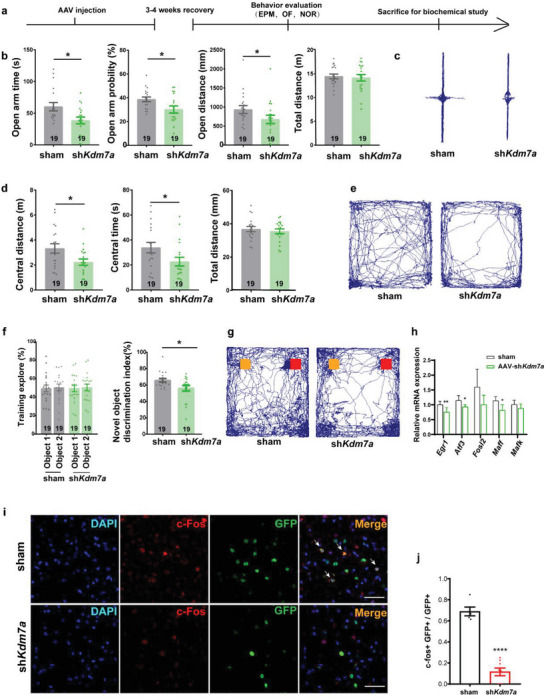
KDM7A affects neuron function in mice. a) Graphic overview of the mouse study. b) The effects of sh*Kdm7a* on anxiety‐like behaviors of mice in the elevated plus maze test (sham n = 19, sh*Kdm7a* n = 19). The data were presented as the mean ± SEM. T test: *p < 0.05. c) Representative track diagrams of shCtrl and sh*Kdm7a* mice in elevated plus maze test. Horizontal arms were open arms. d) The effects of sh*Kdm7a* on anxiety‐like behaviors of mice in the open field (sham n = 19, sh*Kdm7a* n = 19). The data were presented as the mean ± SEM. T test: *p < 0.05. e) Representative track diagrams of shCtrl and sh*Kdm7a* mice in open field. f) The effects of sh*Kdm7a* on behaviors of mice in the novel object recognition test (sham n = 19, sh*Kdm7a* n = 19). The data were presented as the mean ± SEM. T test: *p < 0.05. g) Representative track diagrams of shCtrl and sh*Kdm7a* mice in novel object recognition. Orange denoted old object, and red denoted new object in test period. h) qRT‒PCR detected IEG expression levels in the hippocampi of the sham and sh*Kdm7a* mice (n = 5). The data were presented as the mean ± SEM. T test for each gene, *p < 0.05; **p < 0.01. i) Representative IF stainings in the CA1 region of the shCtrl and sh*Kdm7a* mouse brains. Arrows indicate GFP+ c‐fos+ cells. Scale bar, 50 µm. j) Quantification of the ratio of c‐fos+ GFP+/GFP+ cells (sham n = 5, sh*Kdm7a* n = 8). The data were presented as the mean ± SEM. T test: ****p < 0.0001.

We made a timeline for the animal study (**Figure** **6**a). Twenty‐one days post AAV injection, behavior tests were performed. Mice were sacrificed after behavior studies. Elevated plus maze (EPM) analysis showed sh*Kdm7a* mice spent less time in the open arms, but there was no significant difference in walking distance compared with that of the sham group (Figure 6b,c). In the open field (OF) experiments, the sh*Kdm7a* mice also showed remarkably less activity in the center part of the chamber but a similar total walking distance compared with that of the sham group (Figure 6d,e). The EPM and OF test results indicated significantly increased anxiety in sh*Kdm7a* mice. Next, novel object recognition (NOR) was performed to evaluate mouse short‐term memory. The sh*Kdm7a* mice showed less interest in exploring new objects than the sham mice but spent similar amounts of time exploring objects during the familiarization phase (Figure 6f,g), which indicated deficits in short‐term memory formation. We sacrificed mice after the behavior test and dissected hippocampus for biological analysis. IEGs were downregulated in the hippocampus (Figure 6h), which was correlated with the in vitro data. *c‐Fos* is the most studied IEG and is widely used as a marker for neuronal activity.^[^ ^11^, ^29^ ^]^ In our study, *c‐fos* expression was markedly decreased in the CA1 region of the sh*Kdm7a* mice (Figure 6i,j), which indicated that neuronal activity decreased after *Kdm7a* knockdown.

## 3 Discussion

**Figure 7:**
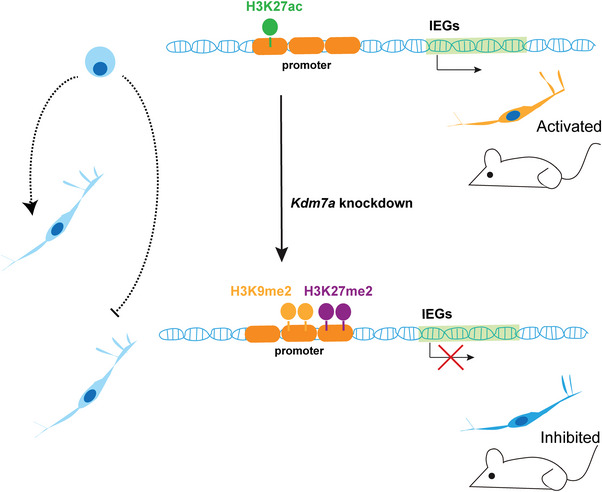
Working model depicting how KDM7A regulates IEGs in mouse neurons. IEGs play a crucial role in maintaining the normal function of neurons. *Kdm7a* knockdown disrupted the balance of histone modifications. Increased H3K9me2, H3K27me2 and decreased H3K27ac at promoters of some IEGs led to the inhibition of neuron differentiation and activity.

In this study, we show that KDM7A targets thousands of genes, affects a variety of histone modifications, and regulates gene expression probably by collaborating with some TFs. Particularly, KDM7A regulates the expression of IEGs, which play important roles in neuronal differentiation and activity (**Figure** **7**).

Although KDM7A is a demethylase that removes H3K9me1/2 and H3K27me1/2 modifications, its dysregulation can also cause changes of other histone modifications. Besides H3K9me2 and H3K27me2, two known substrates of KDM7A, we found that H3K9me3 and H3K27me3 at the TSS, also slightly increased after *Kdm7a* knockdown. H3K9me2 and H3K27me2 are substrates of H3K9me3 and H3K27me3 respectively. It was possible that when the substrate increased, H3K9me3 and H3K27me3 increased. These increased repressive modifications may lead to downregulation of the transcription of some IEGs. It is interesting that our data also showed H3K27ac decreased after *Kdm7a* knockdown. Histone modifications are dynamic equilibrium in cells.^[^ ^30^ ^]^ It was reported that H3K27ac and H3K27me3, acting on the same residue, are mutually exclusive,^[^ ^31^ ^]^ which may give a possible explanation that *Kdm7a* knockdown induce the reduction of H3K27ac on TSS.

We also found genes with KDM7A binding on promoter showing significantly higher expression than other genes, suggesting that KDM7A is relevant to active gene expression. However, there were a nearly equal set of KDM7A target genes that were upregulated versus downregulated when *Kdm7a* was knocked down, suggesting that there could be additional regulating mechanisms besides the demethylase function of KDM7A in regulating gene expression. Furthermore, knocking down *Kdm7a* altered numbers of genes in expression, both with and without KDM7A binding on promoter, suggesting both direct and indirect gene regulation by KDM7A.

Previous studies reported that lysine‐specific demethylases can act as molecular scaffolds to target chromatin modifiers to specific genomic regions. For instance, KDM2B is able to bind to CpG island regions thus recruiting PRC1 to these genomic regions.^[^ ^32^ ^]^ KDM3A and KDM6 demethylases play a role in chromatin remodeling by linking transcription factors with the SWI/SNF chromatin remodeling complex.^[^ ^33^ ^]^ Thus, KDM7A may also have some non‐enzymatic functions. Interestingly, we found that up‐regulated genes show a lower expression level while down‐regulated genes present a higher expression level in shCtrl cells, which indicated that KDM7A may be involved in gene expression regulation by non‐enzymatic function as some TFs do.^[^ ^34^ ^]^ KDM7A was previously reported to act as a transcriptional co‐activator.^[^ ^8^ ^]^ The transcription factors and interactions may be specific to cell type, time point and gene locus.^[^ ^8^ ^]^ Our finding revealed that KMD7A shared binding motifs with multiple TFs, suggesting it may collaborate with TFs to regulate gene expression. It may also explain that genes with KDM7A binding on TSS show higher expression levels may partially due to the AP‐1 binding on promoters. *Fosl2* was found decreased in our study with histone modifications changed, indicated that AP‐1 may be impaired when *Kdm7a* knockdown, which could in turn contribute to genes inhibition. In addition, GO analyses were performed of these up/down‐regulated genes. Down‐regulated genes were much enriched, two terms in which got our attention: negative and positive regulation of transcription from RNA polymerase II promoter, which gave a possible explanation of why a nearly equal set of direct KDM7A target genes are upregulated versus downregulated when *Kdm7a* is knocked down.

According to our results, repressive histone modifications such as H3K9me2 and H3K27me2 significantly increased at TSS when *Kdm7a* knockdown, indicating KDM7A played demethylase role on these loci, which could lead to expression decrease of target genes. In addition, we found KDM7A altered multiple histone modifications on TSS of some IEGs such as *Egr1*, *Atf3*, which were known involved in regulating neuron's function. TFs binding on these down‐regulated IEGs were different from those of up‐regulated genes, which revealed that KDM7A regulated IEGs expression may not only by enzymatic function, but also collaborating with TFs in a non‐enzymatic manner.

The prefrontal cortex and hippocampus play key roles in cognition, memory, and motivation,^[^ ^12^ ^]^ and the expression of IEGs increases with social interaction and social recognition in these brain regions.^[^ ^11^, ^13^ ^]^ IEGs we found be regulated by KDM7A play crucial roles in CNS, thus we gave some introductions of these genes. The protein encoded by *Egr1* was reported involved in an important signaling pathway downstream of ERK/MAPK, which leads to neuronal differentiation.^[^ ^35^ ^]^ Knocking down *Egr1* inhibits neurite growth, while overexpression of *Egr1* promotes neurites in N2A neuroblastoma cells.^[^ ^36^ ^]^ In neurons, the induction of ATF3 expression prevents cell death and promotes the formation and elongation of neurites that enhance neural regeneration.^[^ ^37^ ^]^ MAFF proteins are important players in the mechanisms of cell division and differentiation in PC12 cells.^[^ ^38^ ^]^ FOSL2 forms AP‐1 complexes with FOSL1, Fos‐B, C‐FOS, JUN, JUNB, and JUND, and FOSL2 forms dimers with JUN to activate LIF transcription.^[^ ^39^ ^]^

In this work, functional enrichment assays in KEGG showed that MAPK pathway was negatively enriched when *Kdm7a* knockdown (Figure S1b, Supporting Information). MAPK pathway played important roles in axonal extension and guidance.^[^ ^40^ ^]^ It would be a feasible plan to combine KDM7A and core genes in MAPK pathways to further characterize molecular mechanism in future. It would also be interesting to detect indirect effects such as cell excitability by electrophysiology,^[^ ^41^ ^]^ the technique widely used in neuroscience using mice acute brain slices. Also, Ca^2+^ signals can be recorded in alive mice by fiber photometry,^[^ ^42^ ^]^ which has growing utilized in recent years. These investigations would help to understand potential mechanisms of KDM7A more complete.

In our experiments, additional shRNA targeting on different domain in *Kdm7a* would provide more evidences to complete the effect of KDM7A on neuronal function, which deserve for further investigations. In the current work, *Kdm7a* knockdown in DG of mice was conducted by neuron specific AAV injection. It would be better to perform behavior studies using mice that receive AAV injection which overexpress *Kdm7a*. It's also better to perform IUE to overexpress *Kdm7a* to observe neuron differentiation in embryonic mice.

Epigenetic retulation plays an important role in the nervous system,^[^ ^43^ ^]^ and it is crucial to identify the alteration of histone modifications and the target genes. Our study identified thousands of KDM7A targeted genes, including IEGs, which may provide a foundation for understanding functional roles of KDM7A in neurological development and disorders.

## 4 Experimental Section

### Cells

Mouse Neuro‐2a cells (N2a) (National Collection of Authenticated Cell Cultures, TCM 29) were cultured in DMEM (Thermo Fisher) with 10% fetal bovine serum (Gibco) at 37 °C and 5% CO_2_.

### Lentiviral Infection

N2a cells were seeded onto 6‐well plates one day prior to infection at a density of 1×10^5^ cells/well. Three shRNA lentiviruses were used in this study: sh*Kdm7a*‐1, sh*Kdm7a*‐2 lentivirus, and scrambled shCtrl lentivirus. The following day, polybrene (Shanghai OBiO Technology Corp., Ltd.) was diluted to a final concentration of 5 µg ml^−1^ with DMEM with 10% v/v FBS. The multiplicity of infection (MOI) value used in this study was determined when the efficiency of infection reached 80% 2 days post‐infection. N2a cells were infected at a MOI of 80. After 24 h, the medium was removed and replaced with complete medium. Lentiviruses were purchased from Shanghai OBiO Technology Corp., Ltd. Short hairpin RNA (shRNA) targeting the 3′‐untranslated region (UTR) of mouse *Kdm7a* mRNA was as follows:

shCtrl, 5′‐CCTAAGGTTAAGTCGCCCTCG‐3′,

sh*Kdm7a*‐1, 5′‐GCATCATGCTGTGGACATT‐3′,

sh *Kdm7a* ‐2, 5′‐GCAGGGACATACTTTGTTT‐3′.

Lentivirus of full length *Kdm7a* was obtained from Minghui^[^ ^16^ ^]^ and utilized as described above.

### Cell Differentiation Conditions

In differentiation studies, 10^4^ N2a cells were plated in 6‐well plates 12 h before induction. The medium was replaced by DMEM + 2% fetal bovine serum with 10 µM retinoic acid in dimethyl sulfoxide (DMSO) (Sigma, St Louis, MO, USA). The medium was replaced every day for 4 continuous days. An equal volume of DMSO in DMEM + 2% fetal bovine serum was used in control cells. Cells with neurites at least 1.5 times the length of the cell body diameter were considered differentiated cells. At least 500 cells were analyzed for each group, and the percentage of differentiated cells was quantified.

### Animals and Housing

C57BL/6 mice and E13.5 ICR mice used in this study were obtained from Beijing Vital River Laboratory Animal Technology Co., Ltd. All the C57BL/6 mice were male. Mice were housed with a 12/12 h light/dark cycle and with free access to food and water.

### Reverse Transcription‐Quantitative Polymerase Chain Reaction (RT‐qPCR)

PrimeScript RT Master Mix (TaKaRa RR036A) was used for RNA reverse transcription. Quantitative PCR was conducted with FastStart Essential DNA Green Master on the Roche LightCycler 96 Real‐time System. The relative expression was normalized to β‐actin using the 2^−ΔΔCq^ method. The following thermocycling conditions were used: initial denaturation at 95 °C for 5 min and 40 cycles of 10 sec at 95 °C and 30 s at 60 °C. The primers used in this study were shown in **Table** **1**:

**Table 1:** Primers for qRT‐PCR

Primers	Sequence
*Kdm7a* F *Kdm7a* R *Mtap2* F *Mtap2* R *Syp* F *Syp R* *Egr1* F *Egr1* R *Egr3* F *Egr3* R *Fosl2* F *Fosl2* R *Atf3* F *Atf3* R *Nr4a1* F *Nr4a1* R *Maff* F *Maff* R *Mafk* F *Mafk* R *Actin* F	TTGGCGTGGAAGAGCATCAT AGCTGACTGCCATGCATCTT AACCAATTCGCAGAGCAGGA GCTGCTTAGGAGTAGCTGGG CCACAGCAGTGTTCGCTTTC AGCCTGTCTCCTTGAACACG ACATCAGTTCTCCAGCTCGC TAGAGGGAAACCCCAGTCCC GGGAAGGCTTGGTTGGAGAC GAGCTTCGACGCTTTTGTCC CCTGTCTGCCTTGGTTAGGG TCTACCCGGAACTTCTGCTG GGGTGCACACTATACCTGCT TGTTTCTGAGGTAGGCTGTCA TGAATTGGATGCCCGGGTGA AGCTTGAATACAGGGCATCTCCAC TGAGGATGTGGGCGATGGAT CTCAACTCGCGCTTGACCTTC AGTCGGAACGAGAAGTCCGA CGCCTCCTTCTTGACCTTCAAT TTCCTTCCTGGGCATGGAGT
*Actin* R	TCTTCATTGTGCTGGGTGCC

### Western Blotting

Tissues and cells were lysed in radioimmunoprecipitation assay buffer (Beyotime Institute of Biotechnology, Shanghai, China). The supernatants of centrifuged lysates (14,000 × g for 5 min at 4 °C) were diluted in 5X Laemmli SDS sample buffer (Beyotime Institute of Biotechnology) at a 4:1 ratio. Total protein was quantified using a bicinchoninic acid assay (Beyotime Institute of Biotechnology) and then boiled at 95 °C for 5 min. Proteins were separated by SDS‒PAGE on 10% gels. The separated proteins were transferred onto nitrocellulose membranes (Pall Life Sciences, Port Washington, NY, USA) and incubated with primary antibodies against KDM7A (cat. no. A14692; 1:1,000, ABclonal) and GAPDH (cat. no. 10494; 1:2,000, ProteinTech Group, Inc., Chicago, IL, USA) overnight at 4 °C. After washes with Tris‐buffered saline and Polysorbate 20, the membranes were incubated with horseradish peroxidase‐conjugated secondary antibody (cat. no. A00098; 1:2,000; GenScript Co., Ltd., Nanjing, China) for 2 h at room temperature. The protein‐antibody complexes were visualized using the SuperSignal West Femto Maximum Sensitivity substrate (Thermo Fisher Scientific, Inc.) and exposed to medical X‐ray film (Denville Scientific, Inc., Holliston, MA, USA). Grayscale values were quantified using Multi Gauge software (version 3.0; Fujifilm Dimatix, Inc., Santa Claa, CA, USA).

### Immunofluorescence

N2a cells on coverslips were fixed in 4% paraformaldehyde for 10 mins and washed with PBS three times. Mice brain tissues were fixed in 4% paraformaldehyde for 8 h, Sucrose gradient dehydration, frozen, and sliced into 30mm‐thick slices (8mm‐thick for embryonic mice brain) with a Leica CM1950 sectioning cryostat. Nuclei were stained with DAPI. The primary antibodies used in this study included KDM7A (cat. No. A14692; 1:100, ABclonal), Tuj‐1 (cat. No. MAB1195; 1:100, R&D), GFAP (cat. No. G3893; 1:100, MilliporeSigma), CX3CR1 (cat. No. AF5825; 1:100, R&D), c‐Fos (cat. No. 2250; 1:100, CST). Coverslips with adult mice brain slices were observed using a TCS SP8‐Leica Microsystems confocal microscope (Leica, UK) with a 63×oil objective lens and subsequently analyzed using LAS AF Lite Software (Leica, UK). Coverslips with embryonic mice brain slices were observed using Olympus Slideview VS200 and subsequently analyzed using Olyvia‐3.3. Cells with immunoreaction were defined as “positive” cells. ^[^ ^44^ ^]^ Positive cells were counted by a trained observer who was blind to the treatment of the animals. Three sections from each sample were analyzed and the mean percentage of each sample was calculated. Numbers of samples analyzed in different experiments were stated in Figure legends.

### ChIP and DNA Library Generation

N2a cells (1×10^7^) were collected and fixed with methanol. SDS lysis buffer was added to resuspend the cell pellet. Twenty‐five cycles of sonication were performed for N2a cells. The supernatant was collected in a 50 kDa concentration tube, and SDS was removed from the supernatant with IP buffer. Then, 150 µL of detergent mix and an appropriate amount of antibody (KDM7A, cat. no. A14692; 1:1,000, ABclonal; H3K9me2, cat. no. ab1220; 1:50, Abcam) were added to the remaining liquid and incubated overnight at 4 °C with rotation. Fifteen microliters of protein G beads were added and incubated at 4 °C by rotation for 3 h. Beads were washed and then resuspended in 1% SDS prepared with 50 µL of TE and oscillated at 65 °C and 1500 rpm for 15 minutes. Together with the input, TE buffer was added to bring the volume to 300 µL, 25 µL of PK mix was added, and the mixture was placed in a water bath at 65 °C to unravel crosslinking overnight. DNA was extracted by phenol chloroform. Then 0.5 µL of RNase was added to each sample, and incubated in a 37 °C water bath for 1 h to remove residual RNA. DNA library generation was performed by a VAHTS Universal DNA Library Prep Kit for IlluminaV3 (Vazyme Biotech, ND607). Two independent replicates were performed for each group.

### CUT & Tag Library Generation

CUT & Tag library generation was performed by Hyperactive Universal CUT & Tag Assay Kit for Illumina kit (Vazyme Biotech, TD903). In brief, 1×10^5^ cells were incubated with 10 µL of binding buffer‐washed ConA beads in a 1.5 ml EP tube. Fifty microliters of antibody buffer with 1 µg antibody was added and incubated overnight at 4 °C (KDM7A, cat. no. A14692, 1:50, Abclonal; KDM7A, 1:50, custom made, Abclonal; H3K27me2, cat. no. ab24684, 1:50, Abcam; H3K9me3, cat. no. ab8898; 1:50, Abcam; H3K27me3, cat. no. 39155, 1:50, Active Motif; H3K27ac, cat. no. 39133, 1:50, Active Motif; H3K4me3, cat. no. A2357, 1:50, Abclonal). After two washes with dig‐wash buffer, 50 µL of dig‐wash buffer with 0.5 µL of secondary antibody was added and incubated at room temperature for 1 h. After two washes with dig‐wash buffer, 2 µL of pG–Tn5 was added to 98 µL of dig‐300 buffer. Samples were incubated at room temperature for 1 h and then washed twice with dig‐300 buffer. Ten microliters of 5×TTBL was added to 40 µL of Dig‐300 buffer, and the samples were incubated at 37 °C for 1 h. Then, 100 µL of Buffer L/B, 5 µL of Proteinase K and 20 µL of DNA extract beads were added to the previous sample and incubated for 10 min at 55 °C. After extraction, PCR was performed to amplify the libraries. All libraries were sequenced by Illumina Hi‐Seq Xten or Hi‐Seq 2500 according to the manufacturer's instructions. Two independent replicates were performed for each group.

### ChIP‐Seq and Cut & Tag‐Seq Analyses

The aligned reads to mm10 using bowtie2^[^ ^45^ ^]^ with default parameters. PCR duplicates were discarded, and the uniquely mapped reads were acquired by picard.jar. After the bam files were indexed using SAMtools, BAM files were converted to bigWig files and normalized to RPGC using bamCoverage in deepTools v2.5.5.^[^ ^46^ ^]^ The total read depths per genomic region were calculated by SAMtools bedcov.^[^ ^47^ ^]^

For KDM7A, MACS2^[^ ^48^ ^]^ with board mode was applied on each replicate to call peaks with q‐value < 0.1. Then, peaks of replicates were handled by the Irreproducibility Discovery Rate (IDR) with scaled IDR >290.

### RNA‐Seq

Total RNA samples were prepared with TRIzol reagent (Life Technologies). Ribosomal RNA depletion and strand‐specific libraries were constructed with the Ribo‐Zero Gold rRNA Removal Reagent (Illumina) and NEBNext Ultra II RNA Directional Library Prep Kit (NEB), and all libraries were sequenced on an Illumina HiSeq X Ten system.

For analysis of RNA‐seq data, 150‐bp paired‐end reads was aligned to the mouse mm10 reference using HISAT2 v2.1.0^[^ ^49^ ^]^ with default parameters. Uniquely mapped reads was then extracted using the grep command with the NH:i:1 tag. BAM files were converted to bigWig files and normalized to 1× sequencing depth using bamCoverage in deepTools v2.5.5.^[^ ^50^ ^]^ Gene expression was quantified using transcripts per million (TPM) with StringTie v1.3.3^[^ ^51^ ^]^ with the parameter “‐A”. Three independent replicates were performed for each group.

To identify differentially expressed genes, reads overlapping Ensembl genes were reads using HTSeq^[^ ^52^ ^]^ with the following command: htseq‐count ‐f bam ‐r pos ‐s reverse ‐a 10 ‐t exon ‐i gene_id ‐m union. genes defined showing significant differences with log2 (fold change) > 1 or < ‐1 and Benjamini‒Hochberg corrected p value < 0.05 as cutoff using the R package DESeq2,^[^ ^53^ ^]^ excluding genes with zero coverage in all samples.

GO enrichment analyses on gene sets were performed on the DAVID^[^ ^54^ ^]^ website, and biological process terms were ranked based on Benjamini corrected p values.

### Intrauterine Electroporation (IUE)

C57BL/6 mice with E13.5 embryos were intraperitoneally anesthetized with sodium pentobarbital at a dose of 50 mg kg^−1^, and embryos within the intact uterine wall were exposed after cesarean section. Saline was added dropwise during the operation to keep the embryo moist. The embryo was gently pushed in the uterus closer to the uterine wall, the needle tip was inserted into the lateral ventricle and 1–2 µl of plasmid was injected at a concentration of 4 µg µL^−1^. The dye diffusing into a crescent‐shaped shape indicates successful injection of the plasmid into the lateral ventricles. The BTX830 electroporator was used for operation, with 35 V, a pulse duration of 50 ms, an interval of 950 ms, and a pulse number of 5. After the experiment was completed, the embryos were placed back into the abdominal cavity and surgically sutured. After the operation, the pregnant mouse was placed on a thermostatic pad at 37 °C until awake. The embryos were allowed to grow and develop to E15.5. Sacrificed mice and fetal mouse brains were collected, fixed for 6 h with 4% paraformaldehyde, and then dehydrated with 10%, 20%, and 30% sucrose.

### Single‐Cell RNA‐Sequencing Analysis

The expression matrices and metadata of GSE104323 were downloaded from the Gene Expression Omnibus (GEO) (https://www.ncbi.nlm.nih.gov/geo/) and were read into R with Seurat (4.3.0).^[^ ^26b^ ^]^ The data were processed with standard pipeline of Seurat. Briefly, NormalizeData was run with LogNormalize method and scale.factor of 5000. Then, the Variable features were identified by findVariableFeatures using the vst method and 5000 features. To exclude the effects of sex and stress, sex genes (EHD2, ESPL1, JARID1D, PNPLA4, RPS4Y1, Xist, tsix, Eif2s3y, Ddx3y, Uty, Kdm5d) and stress genes (Rpl26, Gstp1, Rpl35a, Erh, Slc25a5, Pgk1, Eno1, Tubb2a, Emc4, Scg5) were removed. After ScaleData was run on remaining genes, principal components were computed with RunPCA. The first 17 principal components were selected by ElbowPlot and were used for t‐SNE with RunTSNE function. Cell clusters were identified with resolution of 1.0. Cell clusters were further annotated according to the expression of the cell type specific marker genes.^[^ ^26a^ ^]^

### Stereotaxic Microinjection

The mice were anesthetized with sodium pentobarbital at a dose of 50 mg kg^−1^ intraperitoneally. Animals were shaved, and the surgical site was prepped with iodine and ethanol swabs and then mounted on a stereotaxic apparatus (1404, David Kopf Instruments) for brain surgery under aseptic conditions. AAV was purchased from OBiO Technology (Shanghai) Corp.,Ltd. AAV was delivered into the DG (−2.0 mm anterior/posterior (AP), +1.25 mm medial/lateral (ML), and 2.1 mm dorsal/ventral (DV) using a microinfusion pump (Legato 130, KD Scientific, Inc., MA, USA) connected to a 33‐gauge Hamilton microsyringe. The flow rate was calibrated and set to 0.1 µl min^−1^; the injection volume was 500 nl site^−1^. Injector was left in place for 10 mins post‐injection. All experimental procedures occurred 21 days post‐virus injection to allow transduction and expression. Successful virally mediated transduction was confirmed postmortem in animals via IF staining.

### Elevated Plus Maze

The elevated plus maze was used to evaluate anxiety in the mice. It consisted of four arms (30 × 5 cm) connected by a common 5 × 5 cm center area. Two opposite‐facing arms were open (OA), whereas the other two facing arms were enclosed by 20‐cm‐high walls (CA). The entire plus maze was elevated on a pedestal to a height of 82 cm above floor level in a room separated from the investigator. Ambient luminosity was maintained at 60 lx to control the anxiogenic feature of light for rodents. The mouse was placed onto the central area facing an OA and allowed to explore the maze for a single 8‐min session. Between each session, any feces were cleared from the maze, and the maze floor was cleaned with 70% alcohol to remove any urine or scent cues. For each animal, the amount of time spent in the CA and OA and total distance moved were automatically recorded by a video. The data were recorded by a video tracking system (Ethovision, Noldus, Netherlands).

### Open Field

The open field experiment was also an experiment used to assess anxiety in mice, and its principle was similar to that of the elevated cross maze. The open field used in the open field experiment was placed in a separate room from the test subject, and the open field was a square container with a side length of 50 cm and a height of 40 cm. A monitor was installed above the open field to track the movement of the animals. The data were recorded by a video tracking system (Ethovision, Noldus, Netherlands). For the test, mice were placed facing the open field wall and allowed to explore freely for 10 mins. After each mouse was tested, it was returned to the cage and wiped with 75% alcohol, and after the alcohol evaporated, the next mouse was tested. Data analysis based on data recorded by the tracking system was the time of exploration and activity distance of mice in the central area of the open field within 10 mins.

### Novel Object Recognition Test

The novel object recognition memory task has been used to evaluate hippocampus‐dependent memory in rodents through an evaluation of the differences in the exploration time of novel and familiar objects. The testing apparatus was a classic open field (i.e., a PVC square arena, 50 × 50 cm, with walls 40 cm high). The open field apparatus was placed in a part of the room separated from the investigator and was surmounted by a video camera connected to a computer. Testing consisted of three different phases: a habituation phase, a sample phase, and a test phase. Following initial exposure, four additional 10‐min daily habituation sessions were performed for mice to become familiar with the apparatus (50 × 50 × 40 cm) and the surrounding environment. On the fifth day, every mouse was first subjected to the sample phase, in which two identical objects were placed in a symmetrical position from the center of the arena, and the mouse was allowed to freely explore the objects for 8 mins. After a 20‐min delay during which the mouse was returned to its home cage, the animal was reintroduced into the arena to perform the test phase. The mouse was then exposed to two objects for another 5 mins: a familiar object (previously presented during the sample phase) and a novel object, placed at the same location as during the sample phase. The area of exploration was set to ≈2 cm around the object. The data were recorded by a video tracking system (Ethovision, Noldus, Netherlands).

### Statistical Analysis

Unpaired Student's t‐test, One‐way ANOVA and Two‐way ANOVA were performed using GraphPad Prism 5. Data were expressed as mean ± SEM, and *p*‐values < 0.05 were considered to be statistically significant.

### Ethics Approval and Consent to Participate

All procedures involving mice were reviewed and approved by the Animal Care and Use Committees of Fudan University School of Medicine, China (identification number: 20210302‐024 and FE21120).

## Conflict of Interest

The authors declare no conflict of interest.

## Author Contributions

Y.W. and Q.H. contributed equally to this work. B.W. conceived the project. Y.W. carried out the study, performed most of experiments, analyzed the data. Q.H. performed bioinformatics analyses. Y.X. performed some experiments during revision. Z.Z. and B.W. supervised the study. Y.W., Z.Z., and B.W. prepared the manuscript with the input of all authors.

## Supporting information

Supporting Information

## Data Availability

The data that support the findings of this study are available in the supplementary material of this article.
