# Supplementary material for: The Lysine Demethylase KDM7A Regulates Immediate Early Genes in Neurons
Source: Adv Sci (Weinh). 2023 Aug 10;10(28):2301367. doi: 10.1002/advs.202301367 (PMC10558696; doi:10.1002/advs.202301367)
Supplement: Supplementary file 1 — Supporting Information [file ADVS-10-2301367-s001.pdf]

ADVANCED SCIENCE
Open Access

Supporting Information

for *Adv. Sci.*, DOI 10.1002/advs.202301367

The Lysine Demethylase KDM7A Regulates Immediate Early Genes in Neurons

Yifan Wang, Qin Hong, Yueyue Xia, Zhao Zhang and Bo Wen**

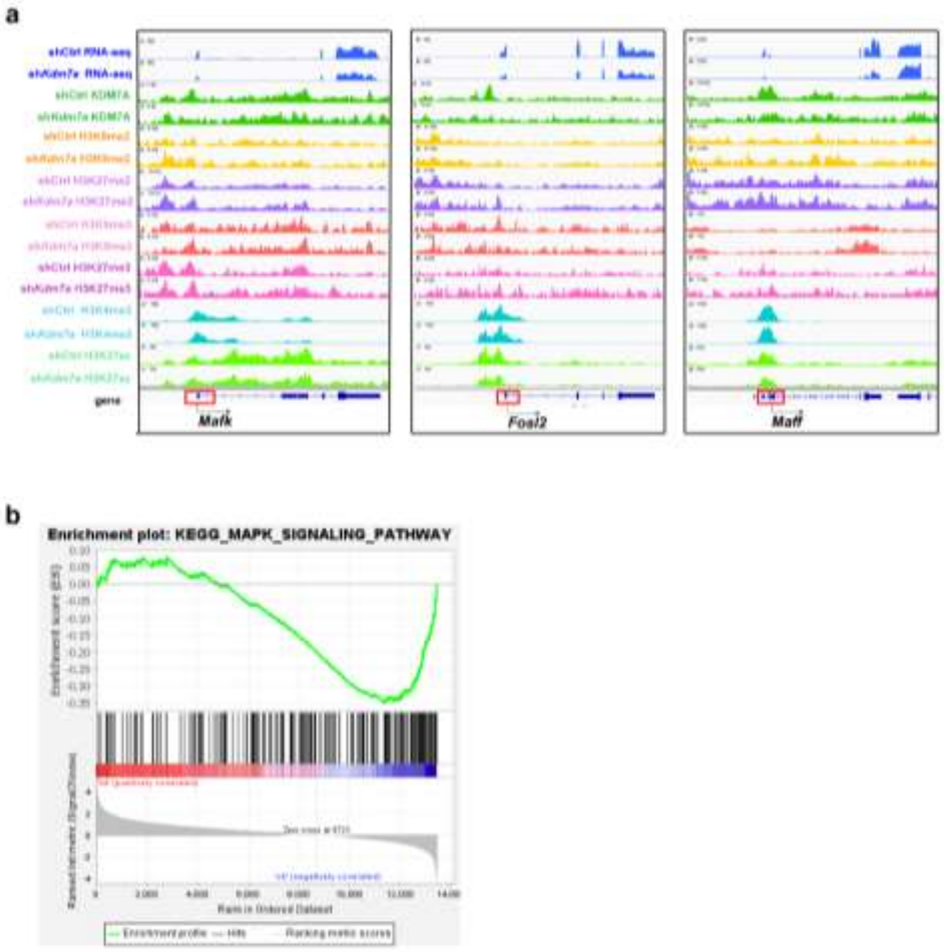

Supplemental Figure 1. IGV tracks and GSEA of KDM7A mediated genes. a) IGV tracks illustrated RNA-seq, KDM7A binding site and histone modifications binding sites at selected gene loci. b) GSEA of KDM7A mediated genes in MAPK signaling pathway.
